# Normal and abnormal aging in bilinguals

**DOI:** 10.1590/S1980-57642009DN20400002

**Published:** 2008

**Authors:** Alfredo Ardila, Eliane Ramos

**Affiliations:** Florida International University, Miami, Florida, USA

**Keywords:** bilingualism, normal aging, cognition, dementia

## Abstract

Bilinguals use two different language systems to mediate not only social communication, but also cognitive processes. Potential differences between bilinguals and monolinguals in task-solving strategies and patterns of cognitive decline during normal and abnormal aging have been suggested.

**Main contribution:**

A research review of the area suggests that normal aging is associated with increased interference between the two languages and tendency to retreat to a single language. General cognitive functioning has been found to be higher in demented bilingual patients if communication is carried out in L1 rather than in L2. Recent research has reported that bilingualism can have a protective effect during aging, attenuating the normal cognitive decline associated with aging, and delaying the onset of dementia.

**Conclusions:**

Regardless of the significant heterogeneity of bilingualism and the diversity of patterns in language use during life-span, current research suggests that bilingualism is associated with preserved cognitive test performance during aging, and potentially can have some protective effect in dementia.

## Abstract (pt)

Pessoas bilingües usam dois sistemas linguísticos distintos na mediação da comunicação social e de processos cognitivos. Em consequência, podem usar estratégias particulares na solução de tarefas específicas, assim como podem passar por processos distintos de declínio cognitivo no envelhecimento normal ou anormal.

**Contribuição:**

Uma revisão da literatura da área sugere que o envelhecimento normal está relacionado com interferência progressiva entre as duas linguas e tendência a retroceder ao uso exclusivo de uma lingua. Em alguns estudos, pacientes bilingües com demência demonstram melhor função cognitiva na comunicação em L1 do que em L2. Em investigações recentes, o bilingüismo tem mostrado um efeito protetor no envelhecimento, atenuando o declínio cognitivo normal e retardando o princípio da demência.

**Conclusões:**

Apesar da grande heterogeneidade presente em populações bilingües e da diversidade de padrões de uso das duas linguas em diferentes estágios de vida, estudos recentes sugerem que o bilingüismo está relacionado a melhores resultados em testes de cognição em idade avançada e, potencialmente, a um efeito protetor na demência.

Language is the major instrument of cognition. Language mediates not only the social relationship systems, but also the control of cognitive processes (“metacognition”).^1^

Bilinguals use two different language systems to mediate both social communication and cognitive tasks. This means that bilinguals are exposed to extra cognitively-demanding tasks, such as language selection and language switching. Potential differences from monolinguals in task-solving strategies and patterns of cognitive decline during normal and abnormal aging may be anticipated.

Brain organization of language may be partially different for the first (L1) and the second language (L2). Functional studies have supported that there is an only partially coincidental pattern of activation for both languages; usually L2 activates a more extended brain system, but the differences between the L1 and L2 are related to the mastery of L2, the age of acquisition, and the functional distance between them.^2-6^ Language disturbance patterns associated with brain pathology can be diverse for L1 and L2 depending upon different variables,^7-10^ but most frequently aphasia in L1 and L2 are parallel when both languages have been acquired early in life, and dissociated when L2 has been acquired late in life.

## Bilingualism during normal aging

The pattern of language use frequently changes throughout lifespan.^11-12^ The two languages can be associated with different social contexts and living situations. The second language can be acquired late in life and be related with working activities or migration. L1 and L2 may be used simultaneously only during certain periods of life. The two languages can also be acquired early in life and maintained in active use throughout the whole lifetime. Indeed, a wide variety of alternative patterns of use of L1 and L2 can be found during lifespan.

Diverse cognitive changes, including changes in language abilities, are observed during aging.^13^ It has been proposed that aging and general cognitive decline may have a detrimental effect on the ability to use two different languages. For instance, aging has been associated with increased interference between the two languages.^14^ Interference has been defined as the transference of elements of one language to another at various levels (phonological, grammatical, lexical and orthographical).^15^ Alternation, or code switching, between languages occurs commonly amongst bilinguals and may take a number of different forms, including alternation of sentences and phrases from both languages one after the other, and switching within long narratives.^16^ The bilingual has to select the appropriate language and to keep conversation within it. The bilingual also has to be able to switch between languages when required. The ability to select the appropriate language and switch between languages involves general executive processing and is regarded as a linguistic ability dependent upon frontal lobe activity.^17^ With aging, this ability to select the appropriate language and make the correct switching when required decreases. The result is an increased mixture of both languages.

Notably, bilinguals encode and retrieve certain autobiographical memories in one language or the other according to the context of encoding, and these linguistic characteristics are stable properties of those memories over time.^18^ Consequently, memory tests evaluating long term and autobiographical memory should take into consideration the specific language in which specific memories were acquired.

Rosselli et al.^19^ analyzed the influence of bilingualism on cognitive test performance in older adults. They administered verbal fluency and repetition tests to Spanish/English bilinguals. Verbal fluency was tested by eliciting a verbal description of a picture and by asking participants to generate words within phonemic and semantic categories. Repetition was tested using a sentence-repetition test. Results demonstrated equal performance of bilingual and monolingual participants in all tests except that of semantic verbal fluency. Bilinguals who learned English before age 12 performed significantly better on the English repetition test and produced a higher number of words in picture description than the bilinguals who had learned English after age 12. Other cognitive tests have been used with bilingual populations. For instance, Gollan et al.^20^ administered the Boston Naming Test to 29 Spanish-English bilinguals (mean age=74.0), first in their dominant language and then in their less-dominant language. Bilinguals with similar naming scores in each language, or relatively balanced bilinguals, named more pictures correctly when credited for producing a correct name in either language. Balanced bilinguals also named fewer pictures in their dominant language than unbalanced bilinguals, and named more pictures correctly in both languages if the pictures had cognate names (e.g., dart/dardo). The authors concluded that bilinguals’ ability to name pictures reflects their experience with word forms in both languages. Rosselli et al.^21^ analyzed the performance of Spanish-English bilinguals on the Stroop Test. Participants consisted of 71 Spanish-English bilinguals, 40 English monolinguals, and 11 Spanish monolinguals. No significant differences were observed in color reading but bilinguals performed worse in the color naming condition. No significant differences were observed in the color-word condition. The authors suggested that interference between both languages could account for the differences observed in the naming condition.

Interestingly, histopathological changes in the brain of bilingual individuals have also been reported. Mechelli et al.^22^ found that learning a second language increases the density of grey matter in the left inferior parietal cortex and that the degree of structural reorganization in this region is modulated by the proficiency attained and the age at acquisition.

## Dementia in bilinguals

Communication abilities in bilingual demented patients, and pattern of language decline for L1 and L2 in dementia, are issues rarely mentioned in the dementia literature. It is well known, however, that the ability to maintain fluency in more than one language decreases with aging.^12^ With advancing age, people may tend to retreat to a single language, regardless of a life-long history of bilingualism. L2 is frequently associated with active working life, while retirement is often associated with moving to a more limited familiar environment. Moreover, older bilinguals may experience increased difficulties handling two different languages due to the effects of cross-language interference. These effects in aging bilingual persons can be further exacerbated in those who develop dementia.

Mendez, Perryman, Ponton, and Cummings^23^ studied 51 patients who reported routine use of another language, as well as varying fluency in English. Fifteen of the patients presented with probable Alzheimer’s disease, 16 possible Alzheimer’s disease, nine vascular dementia, five frontotemporal dementia, and six “other dementia or mixed dementia”. All patients were regularly exposed to English as a second language after age 13. Usually they used the first language with family and friends, and used at least some English outside the home. Despite patients’ differences in educational level (mean=10.18; SD=4.93), age at acquisition of English, frequency of use, and baseline fluency in English, all caregivers reported a greater preference of the patients for their original language and decreased conversation in English. Patients presented an evident tendency for words and phrases from the native language to intrude into English conversational speech. The authors found that bilingual dementia patients tended to present asymmetrical language impairment with preferential preservation and use of the first acquired language. They suggested that in dementia, recently learned information is retained the least and older, more remote information is often relatively preserved, consistent with a regression toward the predominant use of the patient’s earliest language. According to Mendez and colleagues^23^, a retreat to the original language in dementia could result from an exacerbation of the cross-language difficulties that typically increase with age. People who are bilingual never totally deactivate either of their two languages, and this can result in interference or intrusions, particularly from the dominant language into the other. Dementia patients tend to mix languages, and have specific problems with language separation.

Meguro et al.^24^ studied four bilingual Portuguese/Japanese patients with Alzheimer’s disease. Different levels of the oral and written language were analyzed in both languages. It was found that all patients presented a decreased naming ability in both languages. Oral reading ability was most impaired in the case of Kanji, followed by irregular words in Portuguese; reading regular words was near normal in both languages: They could recognize and pronounce Kana perfectly, and regular Portuguese words almost perfectly. Three of the patients were diagnosed as having anomic aphasia in both languages, whereas in the severest case the patient was classified as having anomic aphasia in Portuguese and Wernicke aphasia in Japanese.

General cognitive functioning has been found to be higher in demented patients if communication is carried out in L1 rather than L2. Ekman et al.^25-26^ studied demented persons who were born in Finland and had migrated to Sweden (Finnish-Swedish bilinguals). They found that many of these Finnish immigrants had difficulties communicating with their Swedish-speaking caregivers, while their communication with a Finnish-speaking caregiver was adequate. The frequent misunderstanding of a person’s message often leads to one-way communication, in which the caregiver commands and interrupts the demented person. The demented Finnish immigrants functioned on a level of manifest competence that seemed far below their level of latent competence. The authors concluded that the presence of Finnish-speaking caregivers is an environmental change that would markedly enhance the demented Finnish immigrants’ performance and quality of life and also reduce the costs of their care.

Mendez, Saghafi, and Clark^27^ studied two polyglot patients with semantic dementia. The first case was a 71-year-old man who experienced a slow, progressive loss of his ability to use and understand Spanish and German. The patient was a language teacher who had been fluent in Spanish and used it daily in his everyday work. Confrontational naming in English was decreased. The patient had great difficulty understanding even common nouns in Spanish, and was no longer able to understand any German words. On an aphasia battery, word comprehension was moderately impaired in English, and severely impaired in Spanish and German. Words that were comprehended in Spanish or German were not consistently comprehended in English. His magnetic resonance imaging (MRI) studies showed anterior temporal atrophy, greater in the left than the right. The second case was a 66-year-old man who had a 2-year history of progressive loss of the meaning of words and inability to retrieve words. Although Spanish was his first language, he spoke English at work and also knew some Polish. His examination was intact except for naming and recognizing famous faces. Confrontational naming in Spanish was impaired. He could not name pictures of items and made some semantic errors (e.g., “zero” for “circle”). His performance was worse in English than in Spanish, and his Polish was lost. If he comprehended a word in one language, he did not necessarily comprehend it in the other language. The MRI scan showed left anterior temporal atrophy. The authors concluded that in multilingual patients with semantic dementia, semantic anomia was progressively more impaired in their second and third languages compared to their primary languages. Words named and comprehended in one language were not consistently named and comprehended in other languages they could speak. These findings were interpreted as consistent with separate lexical semantic systems for each language.

Filley et al.^28^ reported a case of primary progressive aphasia in a bilingual English/Chinese woman; at the age of 70 she developed anomia that progressed to aphasia. At the age of 76 functional neuroimaging disclosed mild left temporoparietal hypometabolism. Language testing revealed conduction-like aphasia that was comparable in the two languages, although English was slightly better preserved. It was concluded that primary progressive aphasia had disrupted the 2 languages in a similar manner, suggesting their close neuroanatomic relationship in this case.

Usually, the difficulty to select the appropriate language observed in aging bilinguals becomes more severe in cases of dementia. It has been suggested that bilingual speakers with dementia, even in the early stages of deterioration, make errors in selecting the appropriate language and maintaining the correct language during conversational speech.^12,28^ There is, however, a large variability in the extent of inappropriate language use, with some individuals showing more language mixing than others.^19^ Hyltenstam and Stroud^12^ described two cases of Alzheimer’s disease in bilinguals. In one of them, the major problem lay mainly in the area of language choice, whereas in the other the major difficulties were observed in the ability to separate the languages. De Santi et al.^29^ concluded that the ability to make the correct language choice and keep languages separated is correlated with the overall stage of dementia. The mixing may be so significant, that it is not easy to recognize what language the patient is attempting to speak.

It is interesting that normal bilinguals can use the knowledge of two languages to increase verbal production, whereas dementia patients are unable to profit from the knowledge of two different languages. De Picciotto and Friedland^31^ studied verbal fluency abilities in 30 normal aging English-Afrikaans bilingual speakers, and six bilingual subjects with Alzheimer’s disease. A semantic verbal fluency task (animals) was administered in the bilingual mode involving both Afrikaans and English. There was no significant difference between monolingual and bilingual performance. It was observed that some normal bilingual subjects used code switching as a strategy; there was however, no relationship between age of acquisition, pattern of use and verbal fluency scores. In comparison, subjects with Alzheimer’s disease did not make use of code switching strategies, and there was a relationship between age of acquisition, pattern of use and verbal fluency scores. Therefore, it was concluded that normal bilinguals can draw on both languages in an attempt to improve performance whereas demented patients were unable to use this strategy.

Over recent years Bialystok and colleagues^32-38^ have reported several studies supporting that bilingualism can have a protective effect during aging, attenuating the normal cognitive decline associated with aging, and delaying the onset of dementia. In 2004 Bialystok and colleagues published an impacting and influential paper,^32^ suggesting that bilingualism can reinforce executive functions, offsetting the negative effects of aging. They compared the performance of monolingual and bilingual middle-aged and older adults on an executive function test (Simon task). Bilingualism was associated with smaller Simon effect costs for both age groups; bilingual participants also responded more rapidly to conditions that placed greater demands on working memory. In all cases the bilingual advantage was greater for older participants. The authors proposed that control processing is carried out more effectively by bilingual individuals. They further suggested that bilingualism helps to offset age-related losses in certain executive processes. In a later study, Bialystok, Craik and Freedman^35^ selected 184 patients diagnosed with dementia in a memory clinic; about half of them were bilinguals. It was found that bilingual patients showed symptoms of dementia 4 years later than monolinguals, while all other measures proved equivalent. Additionally, the rate of decline in Mini-Mental State Examination (MMSE) scores over the 4 years subsequent to the diagnosis was the same for a subset of patients in the two groups, suggesting a shift in onset age with no change in rate of progression.

In spite of the importance of the issues, with the exception of Bialystok and colleagues’ research, just a few studies have analyzed the potential protective effect of bilingualism during normal and abnormal aging. Kavé et al.^39^ examined a large population of elders, composed of 814 individuals with a mean age of 83 years. Data were drawn from a representative sample of the oldest Israeli Jewish population. These subjects were initially studied in 1989 and during the following 12 years two follow-up studies were performed. Regression analyses showed that the number of languages spoken contributed to the prediction of cognitive test scores beyond the effect of other demographic variables, such as age, gender, place of birth, age at immigration, or education. Mohamed et al.^40^ examined the functioning of inhibitory mechanisms in younger and older bilinguals using a bilingual version of the Stroop test. They found that older bilinguals were slower when they responded in their non-dominant language. Furthermore, older unbalanced bilinguals showed greater interlingual interference when they responded in their second language to visual stimuli written in their dominant language. Balanced bilinguals showed equivalent interference effects under all conditions. Departing from these results, the authors suggest that manipulating two languages may enhance the efficiency of inhibitory mechanisms.

## Conclusions

Bilingualism is a heterogeneous phenomenon and patterns of language use throughout life-span are diverse. Research has supported the assumption that using two or more different languages can frequently provide not only some social but also cognitive advantages. Life experiences may be associated with the use of a specific language, and life memories are also associated with the contextual language existing when the memory was acquired. Seemingly, during normal aging there is a more rapid decline of L2 than L1, and elders tend clearly to prefer using L1. In case of dementia, the cognitive defect is more severe when the demented patient is tested in L2 than when tested in L1. Similarly, psychiatric symptomatology can be non-coincident when the patient is tested using L1 and L2. Recent research has supported the suggestion that bilingualism can have a protective effect during age-associated cognitive decline, and may even delay the onset of the dementia process.
